# Demographic, socio-economic and behavioural correlates of BMI in middle-aged black men and women from urban Johannesburg, South Africa

**DOI:** 10.1080/16549716.2018.1448250

**Published:** 2018-08-06

**Authors:** Lisa K. Micklesfield, Juliana Kagura, Richard Munthali, Nigel J. Crowther, Nicole Jaff, Philippe Gradidge, Michèle Ramsay

**Affiliations:** a MRC/Wits Developmental Pathways for Health Research Unit, Department of Paediatrics, Faculty of Health Sciences, University of the Witwatersrand, Johannesburg, South Africa; b Sydney Brenner Institute for Molecular Bioscience and Division of Human Genetics, Faculty of Health Sciences, University of the Witwatersrand, Johannesburg, South Africa; c Department of Chemical Pathology, National Health Laboratory Service, Faculty of Health Sciences, University of the Witwatersrand, Johannesburg, South Africa; d Centre for Exercise Science and Sports Medicine, Faculty of Health Sciences, University of the Witwatersrand, Johannesburg, South Africa

**Keywords:** BMI distribution across African communities, Obesity, overweight, physical activity, socio-economic status, BMI, South Africa

## Abstract

**Background**: There is a high and increasing prevalence of overweight and obesity in South Africans of all ages. Risk factors associated with overweight and obesity must be identified to provide targets for intervention.

**Objective**: To identify the demographic, socio-economic and behavioural factors associated with body mass index (BMI) in middle-aged black South African men and women.

**Methods**: Data on demographic and socio-economic factors were collected via questionnaire on 1027 men and 1008 women from Soweto Johannesburg, South Africa. Weight and height were measured and BMI was determined. Behavioural factors included tobacco use and consumption of alcohol, and physical activity data were collected using the Global Physical Activity Questionnaire. Menopausal status was determined for the women, and HIV status was available for 93.6% of the men and 39.9% of the women.

**Results**: Significantly more women were overweight or obese than men (87.9 vs. 44.9%). Smoking prevalence (current or former) and minutes spent in moderate to vigorous intensity physical activity was significantly different between the sexes (both p < 0.0001). In the final hierarchical model, marital status (+ married/cohabiting), household asset score (+), current smoking (-), moderate to vigorous physical activity (-) and HIV status (- HIV infected) significantly contributed to 26% of the variance in BMI in the men. In the women, home language (Tswana-speaking compared to Zulu-speaking), marital status (+ unmarried/cohabiting), education (-), current smoking (-) and HIV status (- HIV infected) significantly contributed to 14% of the variance in BMI.

**Conclusions**: The sex difference in BMI and the prevalence of overweight and obesity between black South African men and women from Soweto, as well as the sex-specific associations with various demographic, socio-economic and behavioural factors, highlight the need for more tailored interventions to slow down the obesity epidemic.

## Background

Various studies on black adult South African populations over the last 20 years have reported a high and increasing prevalence of risk factors for cardiovascular disease (CVD) [1–4]. In a study on urban-dwelling black South Africans, it was suggested that the rapid rise in type 2 diabetes (T2D) prevalence is strongly related to higher adiposity levels, as more than 80% of the diabetic participants were either overweight or obese, and also had higher measures of abdominal adiposity compared to the non-diabetic participants [2,5]. The most recent national statistics on the prevalence of overweight and obesity in South Africa have reported that 64% of adult women and 30.7% of adult men are overweight or obese, with the numbers differing quite significantly between the ethnic groups [6,7]. Obesity trends in Africa between 1980 and 2014 have shown an increase in age-standardised mean body mass index (BMI) from 21 kg/m^2^ to 23 kg/m^2^ in men, and from 21.9 kg/m^2^ to 24.9 kg/m^2^ in women [6,8]. These data collated by the NCD Risk Factor Collaboration (Africa Working Group) report a mean BMI higher than the global average in northern and southern Africa and lower in central, eastern and western Africa, with the mean BMI across the five regions generally being higher in women than men.

Several sociocultural, environmental and behavioural determinants of obesity have been identified in black South African women [2,5]; however, it is uncertain if these factors predict BMI in black South African men. The sex difference in obesity prevalence in Africa [6,7] is not observed in high-income countries where obesity is more similar between the sexes [6,8]. Differences in socio-demographic factors and lifestyle behaviours between black South African men and women have been explored in several population-based studies [9,10]; however, whether these factors are interrelated or independent of each other and other potential confounders, and how they are associated with BMI, requires further study.

South Africa is at the forefront of the nutrition transition in sub-Saharan Africa (SSA) [11] and a greater understanding of the determinants of obesity in a rapidly transitioning urban South African setting may be critically important in informing interventions in earlier stages of this transition elsewhere in Africa. Therefore, the aim of this study is to identify the demographic, socio-economic and behavioural factors associated with BMI in a sample of middle-aged black South African men and women.

## Methods

### Design and study participants

Data collection for this survey, as part of the AWI-Gen (Africa Wits-INDEPTH partnership for Genomic Research) [12], took place between 2011 and 2015 on black South African men (n = 1027) and women (n = 1008) residing in Soweto, South Africa. The women were randomly recruited from caregivers of the Birth to Twenty Plus (Bt20+) cohort, the largest longitudinal birth cohort on childhood development and health in Africa to date [13]. The men were randomly recruited from the same communities as the women in Soweto and age-matched. All participants were invited to the research institute for data collection and the same instruments, tools and protocols were used for both men and women. As the women were part of Bt20+, in order to minimise redundancy, home language, education, household assets and marital status data were obtained from data already collected on the Bt20 caregivers at previous data collection time points. The detailed data collection approach for the AWI-Gen study is described in the accompanying paper in this special issue [14].

### Demographic and socio-economic factors

Home language was selected from a list of 12 local South African languages with the option to specify ‘other’ if appropriate. Marital status was categorised as (i) either being married or cohabiting with a partner, or (ii) unmarried, which included being single, divorced or separated, or widowed. Men were asked to report their highest level of education which was categorised as (i) no formal or primary education, (ii) secondary or (iii) tertiary education (three categories). Years of education was collected in the women and categorised as follows (i) ≤ 7 years = no formal/primary education; (ii) 8–9 years = some secondary education; (iii) 10–12 years = secondary education; (iv)>12 years = tertiary education. Employment was categorised as (i) employed (self-employed, formal full-time or part-time employment by someone else, informal employment) or (ii) unemployed. Household assets, a proxy for socio-economic status, were a count of the major household amenities in the household, in working order, at the time of data collection.

### HIV status

A voluntary HIV antibody test, Alere Determine^TM^ HIV-1/2 (Alere San Diego, Inc. San Diego, CA), was offered to all participants and the process included pre- and post-test counselling sessions. The sensitivity of this test is 100% and the specificity is 99.23% (antibody) and 99.66% (antigen). Those who reported that they were HIV positive were not retested. If positive on testing during the recruiting phase, participants were referred to local HIV clinics for a confirmatory serological test, CD4 count and further care.

### Behavioural factors

Tobacco use was evaluated by asking participants if they had ever been exposed to tobacco through smoking or taking snuff. Snuff is a smokeless tobacco that is either inhaled through the nose or through the mouth, or placed on the lip. Smoking was categorised as current smoking, never smoked, or ever smoked, while participants were only required to respond if they used snuff or not. Alcohol consumption was categorised as yes or no in the men only. The Global Physical Activity Questionnaire (GPAQ), developed for global physical activity surveillance, was completed via interview to obtain self-reported physical activity [15]. Total moderate to vigorous intensity physical activity (MVPA) in minutes per week (mins/wk) was calculated by summing occupation, travel-related and leisure-time moderate and vigorous intensity physical activity. Sitting time (hours/day) was used as a proxy for sedentary behaviour. The MVPA (mins/wk) was reported for all participants who completed the GPAQ (n = 1027 men; n = 1008 women), while travel-related physical activity and sedentary time are reported only for those who participated in these behaviours (travel-related physical activity: n = 923 men; n = 711 women, and sedentary time: n = 1027 men; n = 963 women). The MVPA was categorised into none (0 mins/wk), insufficient physical activity (0–150 mins/wk) or sufficient physical activity (>150 mins/wk).

### Menopausal status

Menopausal status was determined by date of final menstrual period (FMP). Pre-menopausal women were defined as those with current regular menses, peri-menopausal women as those whose menstrual periods were irregular and where their last menstrual period was within the previous 12 months, and post-menopausal women as those with no menses for more than 12 months. Women who had had a hysterectomy or those using contraceptives were not staged.

### Anthropometry

Weight and height were measured by trained research assistants using a calibrated electronic scale and stadiometer, respectively, for participants wearing light clothes and barefoot. The BMI measures were categorised from the weight and height measures (weight in kg/height in metres^2^). BMI was categorised into underweight (BMI<18.5 kg/m^2^), normal weight (BMI ≥18.5 and <25kg/m^2^), overweight (BMI≥25 kg/m^2^) and obese (BMI≥30 kg/m^2^).

### Statistical analysis

Data compilation and statistical analyses were performed using Stata v.13.0. Student t-test and the Wilcoxon rank test were used to study parametric and nonparametric variables, respectively. The Chi-square test was used for the study of ordinal and categorical variables such as HIV status and education level among others. Bivariate regression analyses with unstandardised beta coefficients were completed between BMI and the various demographic, socio-economic and behavioural factors. All factors that were associated with a p < 0.20 for the men or the women were then entered into a linear hierarchical regression to determine which factors were independently associated with BMI. Collinearity between the variables was checked by calculating the variance inflation factor (VIF) with the highest VIF being recorded at 1.70. A VIF >10 is considered significant collinearity. As BMI was not normally distributed, log BMI was used as the outcome variable. Model 1 consisted of the demographic and socio-economic variables including age, home language (Zulu, Sotho, Tswana or others), marital status (married/cohabiting or not), education level (no formal or primary, secondary and tertiary) and household asset score (some of physical assets). Model 2 then included all the behavioural variables: smoking (yes or no), alcohol intake (yes or no) in men only, and MVPA, and both models were then combined with HIV status (yes or no), and menopausal stage in the women only, to create Model 3. Structural equation modelling (SEM) was performed to determine whether the observed associations from the hierarchical modelling, between demographic and socio-economic factors, and BMI, were mediated by behavioural variables and HIV status. Latent variables for demographic and socio-economic factors (education level, employment status, household assets, age and marital status) and behavioural (alcohol intake, smoking and tobacco snuff use) were constructed and included with MVPA, HIV status and BMI in the SEM model.

## Results

### Sex differences

Demographic and socio-economic factors, behavioural characteristics and anthropometry, for the men (n = 1027) and women (n = 1008) are presented in Table 1. There was no significant difference in age between the sexes, with a median age for the total sample of 49 (44–54) years. The predominant home language spoken by this cohort was Zulu (36.1%). There were significant sex differences in both demographic and socio-economic factors, with the majority (55.6%) of the men being married or cohabiting with a partner compared to 40% of the women. Nearly 15% of the men reported completing tertiary education compared to 9% of the women, while 65.4% of the men were employed compared to 54.9% of the women. The median (IQR) for total household assets, a proxy for household SES, in the men was 12 (10–15) out of a possible 22, and in the women it was 7 (4–9) out of 13.

**Table 1.** Demographic, socio-economic, behavioural factors and adiposity characteristics of Soweto men (n = 1027) and women (n = 1008).

Variables	Men	Women	Total sample	P-value
**SOCIO-DEMOGRAPHIC AND HEALTH STATUS**				
**Age** (years)	49 (44–55)	49 (44–54)	49 (44–54)	0.24
**Home language**, n (%)				
Zulu	405 (39.4)	329 (32.6)	734 (36.1)	ϰ^2^ = 15.43
Sesotho	186 (18.1)	187 (18.6)	373 (18.3)	0.01
Tswana	134 (13.1)	121 (12.0)	255 (12.5)	
Other	302 (29.4)	371 (36.8)	673 (33.1)	
**Marital status**				
Married/cohabiting	571 (55.6)	266 (40.0)	837 (49.5)	ϰ^2^ = 39.55
Unmarried	455 (44.34)	399 (60.0)	854 (50.5)	<0.01
**Highest level of education**				
No formal/primary education	125 (12.2)	97 (12.12)		N/A
Some secondary education	N/A	335 (41.88)		
Secondary education	749 (72.9)	296 (37.00)		
Tertiary education	153 (14.9)	72 (9.00)		
**Employment**				
Unemployed	353 (34.6)	455 (45.1)	808 (39.9)	ϰ^2^ = 23.29
Employed	666 (65.4)	553 (54.9)	1219 (60.1)	<0.01
HIV status*				
Not infected	764 (79.5)	317 (78.9)	1081 (79.3)	ϰ^2^ = 0.071
Infected	197 (20.5)	85 (21.1)	282 (20.7)	0.789
**BEHAVIOURAL FACTORS**				
**Smoking status**				
Never smoked	309 (30.4)	899 (90.0)	1208 (59.9)	ϰ^2^ = 759.87
Ever smoked	540 (53.1)	49 (4.9)	589 (29.2)	<0.01
Current smoker	168 (16.5)	51 (5.1)	219 (10.9)	
**Use of snuff**				
No	700 (98.3)	810 (80.8)	1510 (88.1)	ϰ^2^ = 520.98
Yes	12 (1.7)	192 (19.2)	204 (11.9)	< 0.01
**PHYSICAL ACTIVITY (PA) AND SEDENTARY TIME**				
Total MVPA (mins/wk)	600 (0–1680)	60 (0–840)	360 (0–1350)	< 0.01
**MVPA categories**				
None (0 mins/wk))	268 (26.3)	458 (45.4)	726 (35.8)	ϰ^2^ = 138.2
Insufficient PA (1–150 mins/wk)	24 (2.4)	83 (8.2)	107 (5.3)	< 0.01
Sufficient PA (≥150 mins/wk)	727 (71.3)	467 (46.3)	1194 (58.9)	
Sedentary time (hours/day)	10 (7–13.3)	9.5 (6.5–12.0)	9.7 (6.7–12.5)	<0.01
Travel-related PA (hrs/week)	1 (0.5–2)	0.5 (0.3–1)	0.8 (0.5–1.5)	<0.01
**ANTHROPOMETRY**				
Height (m)	1.7 (1.7–1.8)	1.6 (1.5–1.6)	1.6 (1.6–1.7)	<0.01
Weight (kg)	70.8 (59.8–83.4)	81.4 (70.6–95)	76.4 (63.8–89.3)	< 0.01
BMI (kg/m^2^)	24.2 (20.6–28.5)	32.8 (28.5–37.5)	28.5 (23.0–33.9)	< 0.01
**BMI CATEGORIES**				
Underweight	106 (10.3)	6 (0.6)	112 (5.5)	ϰ^2^ = 580.71
Normal weight	457 (44.5)	116 (11.5)	573 (28.2)	< 0.01
Overweight	282 (27.5)	211 (20.9)	493 (24.2)	
Obese	181 (17.6)	666 (6.6)	847 (41.6)	
**MENOPAUSAL STATUS**				
Pre-menopausal		356 (36.6)		
Peri-menopausal		142 (14.6)		
Post-menopausal		443 (45.5)		
Cannot stage		32 (3.3)		

Normally distributed data presented as mean (SD), non-normally distributed data presented as median (IQR) and categorical data presented as n (%). MVPA: moderate to vigorous intensity physical activity. Underweight = BMI<18.5 kg/m^2^, normal weight = BMI ≥18.5 and <25 kg/m^2^, overweight = BMI ≥25 kg/m^2^ and obese = BMI ≥30 kg/m^2^. * sample size is only for those for whom HIV status was known. Total sample was n = 2035 but missing data for marital status: men (n = 1), women (n = 343); education: women (n = 208); employment: men (n = 8); smoking status: men (n = 10), women (n = 9); snuff use: men (n = 315), women (n = 6); MVPA: men (n = 8); weight: men (n = 1), women (n = 9); height: women (n = 9); menopausal status: women (n = 35).

Of the 402 women who knew their HIV status, 21% were HIV positive, compared to 20% of the 961 men who knew their HIV status. Just less than 6% (5.9%) of the sample (men and women) had previously been diagnosed with diabetes (self-reported), and menopausal stage in the women was determined as follows: 36.6%, 14.6% and 45.5% for pre-menopausal, peri-menopausal and post-menopausal, respectively, while 3.3% could not be staged. Body mass index was significantly higher (p < 0.0001) in the women than the men, with 87.9% of the women being overweight or obese compared to 44.9% of the men.

Smoking was significantly different between the sexes with the majority of men (69.6%) either being current smokers or ever smokers, while 90% of the women had never smoked. In contrast, significantly more women reported using snuff compared to men (19.2 vs. 1.7%; p < 0.0001). The majority (71.2%) of the men reported consuming alcohol. Alcohol consumption data were not available for the women. Minutes spent in MVPA was significantly higher in the men than women (p < 0.0001), and 71.6% of the males met the physical activity recommendations of 150 minutes of MVPA a week compared to 46.3% of the women. Sedentary time (mins/day) and travel-related physical activity were significantly higher in the men than in women (both p < 0.0001).

### Bivariate analyses with BMI

The bivariate associations between the demographic, socio-economic and behavioural factors, and BMI, are presented in Table 2. Age was significantly associated with BMI in the men only (p < 0.01), while being unmarried was inversely associated with BMI in the men but positively associated with BMI in the women (both p < 0.01). SES factors such as having a tertiary education, being employed and household assets were positively associated with BMI in the men (p < 0.01), but none of these SES factors were associated with BMI in the women.

**Table 2.** Bivariate analyses (unstandardised beta coefficient) of the association between demographic, socio-economic and behavioural factors, and BMI, in Soweto adults.

	Men		Women	
	Coefficient (95% CI)	p-value	Coefficient (95% CI)	p-value
**Age** (years)	0.12 (0.06, 0.17)	**<0.01**	0.02 (−0.06, 0.10)	0.60
**Home language**				
Zulu	Ref	0.32	Ref	0.59
Sesotho	0.50 (−0.48,1.49)	0.90	−0.36 (−1.66, 0.93)	**0.03**
Tswana	−0.07 (−1.18, 1.04)		−1.71 (−3.23, −0.19)	
Other	−0.51 (−1.36, 0.33)	0.24	0.14 (−0.94, 1.21)	0.80
**Marital status**				
Married/cohabiting	Ref	**< 0.01**	Ref	**<0.01**
Unmarried	−2.98 (−3.66, −2.30)		2.57 (1.43, 3.71)	
**Highest level of education**				
No formal/primary	Ref	NA	Ref	0.52
Some secondary	NA	0.70	−0.55 (−2.24, 1.14)	0.65
Secondary	−0.21 (−1.27, 0.85)	**<0.01**	−0.40 (−2.11, 1.32)	0.99
Tertiary	2.99 (1.67, 4.31)		−0.02(−2.29, 2.25)	
**Employment**				
Unemployed	Ref	**<0.01**	Ref	0.26
Employed	1.87 (1.15, 2.59)		0.52 (−0.38, 1.42)	
**Household score**	0.54 (0.43 0.64)	**<0.01**	0.05 (−0.07, 0.18)	0.39
**HIV status**				
Not infected	Ref	**<0.01**	Ref	**0.007**
Infected	−3.12 (−4.02,−2.28)		−2.22 (−3.81,−0.62)	
**Smoking status**				
Never smoked	Ref	**<0.01**	Ref	**0.01**
Current smoking	−4.09(−4.84, −3.35)	0.33	−2.58 (−4.66, −0.52)	0.05
Former smoking	−0.50 (−1.5, 0.50)		−2.04 (−4.07, −0.01)	
**Use of snuff**				
No	Ref	Ref	Ref	0.44
Yes	1.18 (−2.05, 4.41)	0.47	−.45 (−1.59, 0.69)	
Total MVPA (mins/wk)	−0.0007 (−0.01, −0.0004)	**<0.01**	−0.0003 (−0.01, 0.0002)	0.23
**MVPA categories**				
None (0)	Ref	0.80	Ref	0.56
Insufficient PA	−0.30 (−2.64, 2.03)	**< 0.01**	−0.50 (−2.19, 1.19)	0.37
(1–150 mins/wk)	−1.84(−2.63, −1.06)		−0.43 (−1.37, 0.50)	
Sufficient PA				
(>150 mins/wk)				
Sitting (hours/day)	0.02 (−0.04, 0.08)	0.49	−0.002(−0.12, 0.11)	0.98
Travel-related PA (mins/week)	−0.46(−0.71, −0.21)	**< 0.01**	−0.38 (−0.95, 0.18)	0.18
Pre-menopausal			Ref	0.13
Peri-menopausal			1.08 (−.320, 2.49)	0.59
Post-menopausal			−0.28 (−1.29, 0.73)	0.88
Cannot stage			0.20 (−2.41, 2.81)	

When compared to never having smoked, current smoking was inversely associated with BMI in both the men (p < 0.01) and the women (p = 0.01). All physical activity variables including MVPA (mins/wk), sufficient physical activity (≥ 150 mins MVPA/wk compared to 0 mins/wk MVPA) and minutes per week of travel-related PA, were inversely associated with BMI in the men only (all p < 0.001).

Men and women who were HIV positive had a significantly lower BMI than their HIV negative counterparts (p < 0.01 in men, p = 0.007 in women), and self-reported diabetes was associated with a higher BMI in men only (p = 0.001). Menopausal status was not associated with BMI in the women.

### Multivariate hierarchical analyses with BMI

In the first stage of building the hierarchical regression model, all demographic and socio-economic variables with p < 0.20 in the bivariate analyses for either the men or the women were included in the initial multivariable regression model to assess the influence of these factors on BMI (Model 1), for the men and women separately (Tables 3 and 4). For the men, age (p < 0.004), being married or cohabiting (p < 0.01), tertiary education (p < 0.05) and household asset score (p < 0.01) were all positively associated with BMI. For the women, being Tswana-speaking was inversely associated with BMI (p < 0.003), while being unmarried (p < 0.01) and household asset score (p < 0.05) were positively associated with BMI. Based on the R^2^ values for Model 1, 17% of the variance in BMI could be explained by these factors in the men, while 10% could be explained in the women.

**Table 3.** Sex-stratified hierarchical models showing demographic, socio-economic and behavioural factors, and health status, associated with log BMI for men.

	Model 1 Demographic and socio-economic factors n = 1017		Model 2 Demographic, socio-economic and behavioural factors n = 1015		Model 3 Demographic, socio-economic andbehavioural factors, and HIV n = 957	
	Coefficient (95% CI)	p-value	Coefficient (95% CI)	p-value	Coefficient (95% CI)	p-value
**Age** (years)	0.003(0.001,0.005)	**0.003**	0.0023 (0.0003, 0.0043)	**0.026**	0.001 (−0.001, 0.003)	0.350
**Home language**						
Zulu	Ref	0.638	Ref	0.512	Ref	0.606
Sesotho	0.01 (−0.03, 0.04)	0.375	0.01 (−0.02, 0.04)	0.160	1. (−0.03, 0.04)	0.249
Tswana	0.02 (−0.06, 0.02)	0.290	−0.03 (−0.07, 0.01)	0.426	−0.02 (−0.06,0.02)	0.383
Other	−0.02 (−0.02, 0.01)		−0.01 (−0.04, 0.02)		−0.01 (−0.04, 0.34)	
**Marital status**						
Married/cohabiting	Ref	**<0.01**	Ref	**< 0.01**	Ref	**<0.01**
Unmarried	−0.09 (−0.12, −0.07)		−0.07 (−0.09, −0.04)		−0.06 (−0.09, −0.04)	
**Highest level of education**						
No formal/primary	Ref	0.144	Ref	0.255	Ref	0.109
Secondary	−0.03 (−0.07, 0.01)	**0.025**	−0.02 (−0.06, 0.02)	0.089	−0.03 (−0.07, 0.01)	0.342
Tertiary	0.06 (0.01, 0.11)		0.04 (−0.01, 0.10)		0.03 (−0.03, 0.08)	
**Household score**	0.02 (0.01, 0.02)	**<0.01**	0.01 (0.01, 0.02)	**< 0.01**	0.01 (0.01, 0.02)	**<0.01**
**Smoking status**						
Never smoked			Ref	**< 0.01**	Ref	**<0.01**
Current smoking			−0.12 (−0.15, −0.09)	0.580	−0.12 (−0.15, −0.09)	0.433
Former smoking			−0.01 (−0.05, 0.03)		−0.01 (−0.05, 0.02)	
**Consume alcohol**						
No			Ref	0.644	Ref	0.416
Yes			0.01 (−0.02, 0.03)		0.01 (−0.02, 0.04)	
**Total MVPA (mins/wk)**						
			−0.000015 (−0.000025,	**0.003**	−0.00002 (−0.00003,	**0.002**
			−0.000005)		−0.00001)	
**HIV status**						
Not infected					Ref	**<0.01**
Infected					−0.08 (−0.11, −0.05)	
	**R^2^ = 0.17**		**R^2^ = 0.24**		**R^2^ = 0.26**

**Table 4.** Sex-stratified hierarchical models showing demographic, socio-economic and behavioural factors, and health status, associated with log BMI for women.

	Model 1 Demographic and socio-economic factors n = 622		Model 2 Demographic, socio-economic and behavioural factors n = 610		Model 3 Demographic, socio-economic and behavioural factors, and HIV n = 280	
	Coefficient (95% CI)	p-value	Coefficient (95% CI)	p-value	Coefficient (95% CI)	p-value
**Age** (years)	−0.001 (−0.005, 0.002)	0.34	−0.002 (−0.006, 0.001)	0.166	−0.005 (−0.012, 0.001)	0.109
**Home language**						
Zulu	Ref	0.56	Ref	0.708	Ref	0.475
Sesotho	0.01 (0.03, 0.06)	0.003	0.01 (−0.04, 0.06)	**0.01**	−0.02 (−0.09, 0.04)	**0.03**
Tswana	−0.09 (−0.15, −0.03)	0.65	−0.09 (−0.15, −0.04)	0.94	−0.09 (−0.17, −0.01)	0.196
Other	0.01 (−0.04, 0.06)		0.002 (−0.04, 0.05)		−0.04 (−0.11, 0.02)	
**Marital status**						
Married/co-habiting	Ref	**< 0.01**	Ref	**<0.01**	Ref	0.09
Unmarried	0.07 (0.04, 0.11)		0.07 (0.03, 0.10)		0.05 (−0.03, 0.10)	
**Highest level of education**						
No formal/primary	Ref	0.46	Ref	0.39	Ref	**0.017**
Some secondary	−0.02 (−0.08, 0.04)	0.33	−0.03 (−0.09, 0.03)	0.143	−0.11 (−0.19, −0.02)	**0.009**
Secondary	−0.03 (−0.09, 0.03)	0.71	−0.05 (−0.11, 0.02)	0.56	−0.12 (−0.22, −0.03)	0.251
Tertiary	−0.02 (−0.10, 0.07)		−0.02 (−0.11, 0.06)		−0.07 (−0.18, 0.05)	
**Household score**	0.01 (0.00, 0.01)	**0.04**	0.01 (0.00, 0.01)	**0.04**	0.002 (−0.007, 0.011)	0.69
**Smoking status**						
Never smoked			Ref	**0.009**	Ref	**0.013**
Current smoking			−0.11 (−0.20, −0.03)	**0.05**	−0.18 (−0.31, −0.04)	0.420
Former smoking			−0.09 (−0.17, 0.002)		−0.05 (−0.19, 0.08)	
**Total MVPA (mins/wk)**			−0.000005 (−0.00002, 0.00001)	0.55	−0.000014 (0.00, 0.00)	**0.299**
**HIV status**						
Not infected					Ref	**<0.01**
Infected					−0.12 (−0.18, −0.05)	
**Menopausal status**						
Pre-menopausal					Ref	0.350
Peri-menopausal					0.038 (−0.041, 0.117)	0.965
Post-menopausal					0.002 (−0.074, 0.077)	0.990
Cannot stage					−0.001 (−0.171, 0.169)	
	**R^2^ = 0.10**		**R^2^ = 0.10**		**R^2^ = 0.14**	

In the second stage of model building, behavioural factors including smoking, alcohol intake (men only) and physical activity were added to the initial model. In the men, age, marital status and household asset score remained significant in the model, and in addition current smoking (p < 0.01) and minutes per week of MVPA (p < 0.01) were inversely associated with BMI. In the women, the demographic and socio-economic factors in Model 1 remained significant in Model 2, and in addition current smoking was inversely associated (p = 0.01) with BMI. The R^2^ values for Model 2 were 0.24 and 0.01 in the men and the women, respectively.

In the final stage of model building (Model 3), clinical variables were added to the regression model and for both men and women a positive HIV status was inversely associated with BMI (both p < 0.01). Menopausal status was not significantly associated with BMI in the women. The demographic, socio-economic, lifestyle and clinical factors included in the final model were able to explain 26% of the variance in BMI in the men and 14% of the variance in BMI in the women.

In the structural equation model for men, a latent variable for demographic and socio-economic factors had a direct effect on BMI (ß value: −0.0005, 95%CI:−0.001; −0.00001; p = 0.046) but did not have an indirect effect via lifestyle and health factors on BMI (ß value: −0.0001, 95%CI:−0.002; −0.000004; p = 0.06). A similar model could not converge in women.

## Discussion

Recently published data from 245 population-based surveys in Africa have highlighted the increasing prevalence of obesity and its contribution to the rising non-communicable disease prevalence, and highlights the importance of developing strategies for obesity prevention and control [6]. Given the high and increasing prevalence of obesity in Africa as well as South Africa, the aim of this study was to determine the demographic, socio-economic, behavioural and health variables associated with BMI in a cohort of middle-aged black men and women from Soweto, South Africa. We have shown in this study that double (87.9%) the number of women were classified as overweight or obese compared to the men (44.9%) and we were able to explain significantly more of the variance in BMI in the men (26%) compared to the women (14%). In addition, the variance in BMI was explained by direct associations between the various demographic, socio-economic, behavioural and health variables, and BMI, and the lifestyle and health factors did not mediate the effect of demographic and socio-economic factors on BMI.

Life expectancy in South Africa has increased since 2005 with a particularly sharp increase between 2010 and 2011 [16], and may continue to do so with the scale-up of ARV therapy, and the subsequent new guidelines for ARV therapy [17] which include protocols for third-line therapy. Ageing is typically associated with changes in lifestyle behaviours including a reduction in physical activity, an increase in sedentary behaviour, and changes in dietary patterns, all of which may be the result of different environmental and personal factors [18,19]. It is these changes in lifestyle that have typically been seen to be the cause of the increasing BMI that occurs with age [20]. Our study has highlighted both expected and unexpected associations. In the men in this study the positive association between age and BMI was independent of behavioural factors such as physical activity, as well as other demographic and socio-economic factors such as marital status and household asset score. This may be explained by physiological changes occurring with age in men, including decreasing testosterone levels which have previously been shown to be associated with increased body weight, BMI, waist and hip circumferences, and waist-to-hip ratio [21]. In women, changes in sex hormones during the menopausal transition, and a resultant increase in BMI, have been well described [22]; however, there was no difference in BMI between women at different stages of the menopausal transition in previously published data on a subgroup of this cohort [23] and this study did not find a significant association between either menopausal stage or age, and BMI. This may be due to changes in body composition including a decrease in muscle and bone mass, and an increase in fat mass, resulting in no significant change in overall body weight. In women, home language was associated with BMI with Tswana-speaking women having a lower BMI compared to Zulu-speaking women, before and after including behavioural and health factors in the model. In South Africa language represents tribal groups so these differences may be due to sociocultural influences or alternatively genetic differences which will be the focus of future studies.

The inverse association between smoking and BMI has been well described [24], and is confirmed by the results of this study in men and women. There was a significant sex difference in the prevalence of smoking, similar to what has been shown in other studies [25,26], with 90% of the women reporting that they have never smoked compared to 30% of the men. Further, it was the current smokers in both sexes (16.5% in men and 5.1% in women) who had a significantly lower BMI compared to those who have never smoked, while there is no association with former smokers in the final model. The prevalence of smoking in this study is significantly lower than recently released national data on men from the South African Demographic and Health survey in which it was reported that 36% of black adult men smoke tobacco products, but similar in black women who reported a 3% prevalence of tobacco smoking. Although associated with a lower BMI in this study, smoking is a major risk factor for premature mortality [27], NCDs and cardiovascular disease [28], independent of BMI, and is therefore a lifestyle behaviour that must be avoided. Further, smoking has been associated with greater central adiposity [29].

Pisa and Pisa (2017) [30] have shown trend associations between South Africa’s economic growth and adult obesity, and the social patterning of NCD risk factors, including obesity, have been identified in populations at different stages of the epidemiological transition [31]. In this study, socio-economic status as measured by education, employment and household asset score was positively associated with BMI in men; while in women there was an inverse association between SES, as measured by education, and BMI. These associations were independent of behavioural factors including physical activity, smoking and alcohol consumption, and results of the structural equation model confirmed that the association was not mediated by lifestyle. The sex-specific associations highlight the complexity of the association between SES and BMI with studies showing both positive and negative associations in both sexes [10,32,33]. Puoane et al., using data from the South African Demographic and Health Survey, have shown that the association between education and BMI may not be linear in South African women as they showed that women with no formal education and women with tertiary education had a lower BMI than women with some schooling [34]. Other sex-specific associations included that with marital status, as this study has shown that men who were married had a higher BMI than their unmarried counterparts, while married women had a lower BMI than unmarried women. A multi-country study of four SSA countries has shown inconsistent associations between marital status and BMI in the various study settings [35] and suggests the possible influence of population-specific cultural beliefs around body size and marriage.

Physical activity is included as an important component in interventions to prevent and manage obesity, and some South African data support the inverse association between physical activity and BMI, particularly in women [36–38], while other data has shown no association [39]. Recently published data from the PURE study has reported that the majority of participants from urban and rural sites in South Africa were participating in moderate to vigorous intensity physical activity [9], and other studies have shown that the majority of South African adults are meeting physical activity recommendations of 150 minutes of moderate to vigorous intensity physical activity per week [36,39]. In this study, 71% of the men were meeting physical activity guidelines compared to less than half [46.3%] of the women. This represents a significant decrease in physical activity compared to 8 years previously in the same cohort of women (mean age 41 ± 7.84 years), where 67% were classified as physically active [39]. Similarly, median sitting time has increased from 3 hours/day to 9.5 hours/day [39]. The results of this study have shown an inverse association between physical activity and BMI in the men, before and after adjusting for age and various socio-economic and behavioural factors, as well as HIV status. It also confirms the important contribution of travel-related physical activity to health outcomes in this sample of middle-aged black South African men. Physical activity for transport has been identified as an important domain of physical activity in Africa [40], and this highlights the importance of not only examining physical activity volume, but also understanding physical activity patterns in different populations. The reasons for the lack of association between physical activity and BMI in women are unclear but may be due to the lower volume of physical activity, amounting to only one hour per week, in the women in this study.

South Africa faces a multiple burden of disease due to the high prevalence of HIV and other infectious diseases, as well as obesity and co-morbid diseases [41]. Data from the 2014 South African National HIV Prevalence, Incidence and Behaviour Survey reported a national HIV prevalence of 12.2% [42] compared to 20.7% in the current study. The inverse association between HIV infection and BMI has been extensively reported [43], and confirmed by the results for both men and women in this study.

### Strengths and limitations

Although the study is cross-sectional and therefore only associations with BMI can be explored rather than predictors or other causal factors, hierarchical modelling and structural equation modelling were used to determine whether these associations were mediated by lifestyle and health factors. These factors have also only been examined in an urban sample so its generalisability to other contexts is unknown; however, the sample size is large and representative of a community that is further along the epidemiological transition than other communities in Africa, and therefore findings in this study may inform intervention strategies in other transitioning communities. A further limitation of this study was that all the behavioural data including physical activity were collected via self-report and therefore introduce the possibility of recall bias, and no nutritional data were available on these men and women. The use of some historical socio-demographic data for the women must be acknowledged; however, these women had been part of a longitudinal cohort and were from a relatively stable community so it was assumed that these factors would not have changed significantly.

## Conclusions

In conclusion, there was a significant sex difference in BMI and the prevalence of overweight and obesity was significantly higher in the women than the men in this urban South African sample. Although some of the socio-economic factors associated with BMI were the same in the men and the women, there were some sex-specific associations that should be considered in further studies. The possibility of further longitudinal work in this field will provide the opportunity to explore the changes in these, and other, factors at not only the individual, but also the household and community level, thereby providing areas for possible intervention.

## Paper context

It is well accepted that obesity is an increasing problem in South Africa, particularly in black women. This paper provides data on the demographic, socio-economic, behavioural and clinical factors associated with body mass index in men and women, and contributes to our understanding of how these factors may differ between the sexes. Significantly more of the variance in BMI can be explained by these factors in the men, and these can represent targets for future interventions which may need to be sex-specific.
